# Applications of Enteroendocrine Cells (EECs) Hormone: Applicability on Feed Intake and Nutrient Absorption in Chickens

**DOI:** 10.3390/ani13182975

**Published:** 2023-09-20

**Authors:** Jihwan Lee, Woo Kyun Kim

**Affiliations:** Department of Poultry Science, University of Georgia, Athens, GA 30602, USA; jihwan.lee@uga.edu

**Keywords:** enteroendocrine cell, chickens, feed intake, nutrient absorption, gut hormone

## Abstract

**Simple Summary:**

Normal feed intake and efficient nutrient absorption are prerequisites for achieving rapid growth, high body weight and dimensions, high carcass and meat yield, and feed efficiency in chickens. Gut hormones released from enteroendocrine cells (EECs) have been recognized as regulators for appetite as well as nutrient absorption in other species. However, the underlying regulation mechanisms of appetite control and nutrient absorption by gut hormones are not fully understood in chickens. This review suggests that gut hormones released from EECs play important roles in appetite and nutrient absorption, and these hormones are considered to be able to influence the reduction of feed intake by infection (e.g., *Eimeria* spp. and *Salmonella* spp.) and environmental stresses (e.g., heat stress and high stock density).

**Abstract:**

This review focuses on the role of hormones derived from enteroendocrine cells (EECs) on appetite and nutrient absorption in chickens. In response to nutrient intake, EECs release hormones that act on many organs and body systems, including the brain, gallbladder, and pancreas. Gut hormones released from EECs play a critical role in the regulation of feed intake and the absorption of nutrients such as glucose, protein, and fat following feed ingestion. We could hypothesize that EECs are essential for the regulation of appetite and nutrient absorption because the malfunction of EECs causes severe diarrhea and digestion problems. The importance of EEC hormones has been recognized, and many studies have been carried out to elucidate their mechanisms for many years in other species. However, there is a lack of research on the regulation of appetite and nutrient absorption by EEC hormones in chickens. This review suggests the potential significance of EEC hormones on growth and health in chickens under stress conditions induced by diseases and high temperature, etc., by providing in-depth knowledge of EEC hormones and mechanisms on how these hormones regulate appetite and nutrient absorption in other species.

## 1. Introduction

One of the many functions of gut hormones secreted before and after feeding is to give signals to the brain that regulate feed intake [1,2]. Numerous studies have reported that gut hormones (e.g., ghrelin, cholecystokinin, CCK, peptide YY, PYY, glucagon-like peptide-1, and GLP-1) induce substantial change in feed intake [3,4]. In addition, certain gut hormones help to digest feed and absorb nutrients by acting on the endothelial cells and gastrointestinal epithelium [5]. Although the absorption of carbohydrates, protein, and fat has been persistently investigated in many studies, it is not clearly shown how the gut hormones work for nutrient absorption [6,7,8]. Mellitzer et al. [9] and Beucher et al. [10] reported that the absence of enteroendocrine cells (EECs) in mice resulted in failed fat absorption, thereby affecting body weight and survival rate. Likewise, McCauley [5] indicated that loss of all EECs can cause chronic malabsorptive diarrhea. These findings suggest that gut hormones are closely involved in nutrient absorption [11,12]. The understanding of the effects of gut hormones on feed intake regulation and nutrient absorption has been established mostly in humans, rodents, and/or other mammals such as pigs and cattle. However, the physiological roles of gut hormones in poultry differ from those of other species [13]. Therefore, in the current review, we discuss the overall mechanisms of these hormones in controlling feed intake and sensing nutrients in broilers. Additionally, this review provides an overview of the process of the reduction of feed intake and weight gain by several infections (e.g., *Salmonella, Eimeria* spp., and *Clostridium perfringens*).

## 2. Structure of Enteroendocrine Cells Secreting Gut Hormone

### Enteroendocrine Cells

**Figure 1:**
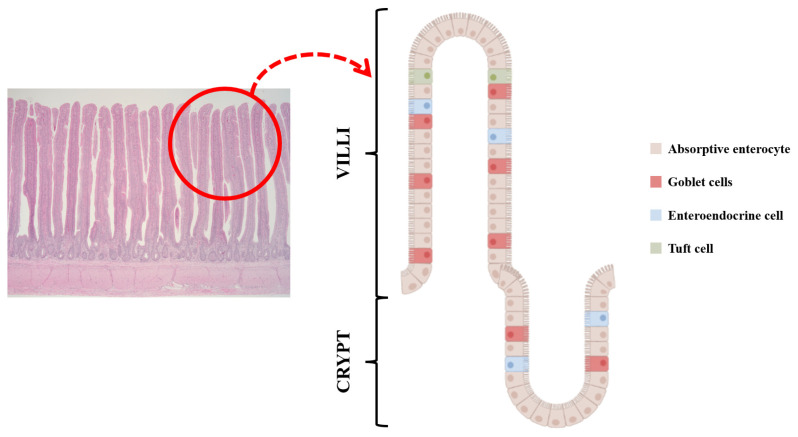
Enteroendocrine cells (EECs) are embedded with other cells such as absorptive enterocytes, goblet cells, stem cells, paneth cells, and tuft cells in intestinal epithelial cells. Along with released hormones, their cell type changes and they are also associated with appetite and nutrient absorption.

Enteroendocrine cells (EECs) are dispersed in villi and crypts throughout the intestinal tract [14,15]. They are located with nonendocrine cells such as absorptive enterocytes, goblet cells, stem cells, and paneth cells (Figure 1) [16] and are also considered the largest endocrine cells relative to the total number of cells, although they occupy less than 1% of the epithelial cell population [16,17]. EECs have been identified and classified by hormone contents (e.g., I cell, K cell, L cell, N cell, S cell, etc.) [18,19]. An overview of the different types of EECs, hormones, and their brief functions are presented in Table 1. The primary function of hormones derived from EECs is to regulate various metabolic responses including interaction with nutrient transporters in brush border and different receptors in the central nervous system (CNS) following feed ingestion [20,21,22]. Therefore, this review provides the mechanisms and effects of gut hormones on feed intake and nutrient absorption according to the hormones released from EECs.

## 3. Feed Intake Control: Mode of Action

### 3.1. Orexogenic Effects

**Figure 2:**
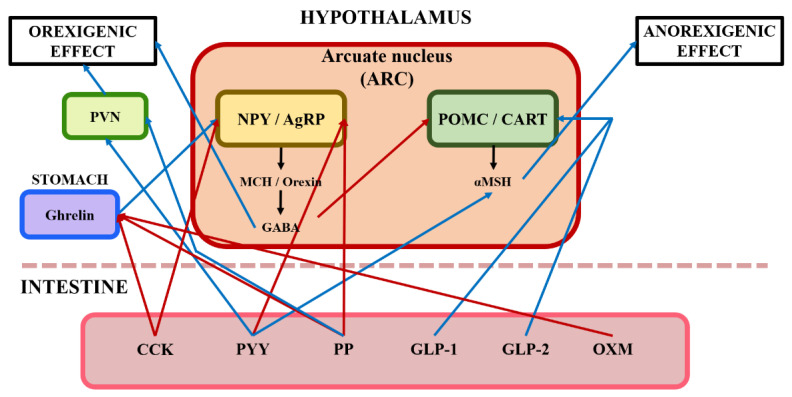
Illustration of the central nervous system's response to gut hormones from enteroendocrine cells in the intestine. Blue arrows represent that it stimulates pathways of orexigenic effect/anorexigenic effect; Red arrows represent that it inhibits pathways of orexigenic effect/anorexigenic effect; CCK, cholecystokinin; PYY, peptide YY; PP, pancreatic polypeptide; GLP, pancreatic polypeptide; OXM, oxyntomodulin; PVN, paraventricular nucleus; NPY, neuropeptide Y; AgRP, agouti-related protein; POMC, pro-opiomelanocortin; CART, cocaine- and amphetamine-regulated transcript; MCH, melanin-concentrating hormone; GABA, gamma-aminobutyric acid; α-MSH, alpha-melanocyte-stimulating hormone.

The mechanism of appetite can mainly be divided into two kinds of effects: orexigenic and anorexigenic effects (Figure 2). The orexigenic effect is related to the increased feed intake and is mitigated by the central and peripheral nervous systems. The arcuate nucleus (ARC) and the lateral hypothalamus (LH) play an important role in appetite regulation. Neuropeptide Y (NPY) and agouti-related protein (AgRP) neurons released from the ARC are called orexigenic neurons [23,24]. The secretion of gamma-aminobutyric acid (GABA) as a neurotransmitter is stimulated by NPY and AgRP, and GABA regulates orexigenic effects by inhibiting anorexigenic neurons [23,25]. Moreover, orexin and melanin-concentrating hormones (MCH) are involved in appetite regulation, although the effect of orexin is inconsistent according to the results of previous studies [26,27] reporting that orexin did not affect the feed intake in broilers.

### 3.2. Anorexogenic Effects

The anorexigenic effect causes feed intake reduction mitigated by central and peripheral nervous systems (Figure 2). Pro-opiomelanocortin (POMC) and cocaine- and amphetamine-regulated transcript (CART) neurons produced from the ARC are called anorexigenic neurons [24,27,28]. Anorexigenic effects have two distinct mechanisms. Firstly, GABA released by NPY and AgRP neurons inhibits the generation of POMC and CART neurons. Secondly, POMC and CART neurons release the alpha-melanocyte-stimulating hormone (α-MSH), leading to feed intake reduction [27].

## 4. Hormones from Enteroendocrine Cells on Feed Intake Control

### 4.1. Ghrelin

Ghrelin is the only known orexigenic gut hormone and hunger hormone [29]. It is an amino acid peptide hormone produced by the EECs in the oxyntic glands in the stomach fundus [30]. Ghrelin binds to G protein-coupled receptors (e.g., growth hormone secretagogue receptor) that are located in areas of the brainstem, the pituitary gland, and the hypothalamus [31,32,33]. During fasting, the ghrelin levels increase nearly two times just prior to meals and then decrease to the lowest value after meals; in addition, ghrelin intravenous injections increase appetite and food intake in humans [34,35]. In rodents, ghrelin injections via intracerebroventricular (ICV) and intraperitoneal (IP) routes also stimulated feed intake and appetite and increased weight gain (Table 2) [29,36]. The possible orectic mechanism of ghrelin is as follows: Feed intake is tightly regulated by the ARC of the hypothalamus [37]. In response to ghrelin binding to growth-hormone secretagogue (GHS) receptors, protein kinase A (PKA) triggers the AMP-activated protein kinase (AMPK). By phosphorylating AMPK, ghrelin stimulates orexigenic NPY and AgRP secretion in the ARC of the hypothalamus [38,39]. As a result, ghrelin increases AMPK and NPY/AgRP and thereby feed intake is increased in mammals. Furthermore, the brainstem, via the vagus nerve, could be a potential extrahypothalamic location to release ghrelin [33]. It has been suggested that the stomach and the nucleus of the solitary tract (NTS) of the brainstem, which has outputs to the ARC, are connected via vagal afferents [33,38,39]. However, the orexigenic effects of ghrelin in avian species have shown controversial results. For example, some studies showed that increased ghrelin levels in blood-induced anepithymia [40], whereas other studies reported that ghrelin injection into intravascular (IV) and IP routes decreased or did not alter feed intake in broilers and laying hens, respectively [41,42,43,44]. The IP injection of 0.5–1 nmol/bird of ghrelin increased feed intake, while 3 nmol/bird and ICV injection of 0.5–1.0 nmol/bird decreased feed intake in Japanese quail [45]. IV administration of acyl-ghrelin at 1 nM/100 g BW/d suppressed feed intake, whereas des-acyl-ghrelin improved feed intake [46]. These findings suggest that these inconsistent outcomes may be attributed to the differences in doses, forms (e.g., acyl- and des-acyl-ghrelin), and injection routes. In addition, different outcomes between mammals and avian species may be due to the activation of AMPK. Xu et al. [47] reported that administration of ghrelin in chickens down-regulated AMPK and decreased appetite. Although all studies mentioned above did not measure AMPK activation and feed intake at the same time, ghrelin injection in chickens showed the opposite results such as reduction of AMPK, unlike studies with other species reporting that ghrelin activates AMPK and then stimulates NPY and AgRP in ARC thereby activating feed intake. However, there is a limitation in previous studies that only estimated the effects of artificial ghrelin injection on feed intake and appetite. Therefore, further research is needed to determine whether endogenous ghrelin suppresses feed intake in avian species.

### 4.2. Peptide YY (PYY)

Peptide YY belongs to the PP fold protein family, which also includes NPY and PP [33]. It is a 36 amino acid peptide and is derived from the L cells of the gastrointestinal tract, particularly those in the colon and rectum [48]. PYY1-36 is divided at the last two amino acids of the N-terminus by DPP-IV, resulting in the truncated form PYY3-36 [49]. However, the major form of PYY is PYY3-36 in animals and humans [50,51]. During fasting, PYY levels are low but reach a peak within a few hours after a meal [48]. The effects of PYY are different depending on the injection location (Table 3). For example, ICV injection of PYY1-36 and PYY3-36 had orexigenic effects [52,53,54,55]. Interestingly, PYY via intra-arcuate (IA) injection showed anorexigenic effects in rodents and humans [51]. At the same time, PYY3-36 injection into IV decreased the feed intake in rodents [51,56]. PYY binds preferentially to the NPY Y2 receptor (Y2R) among the isoform receptors (e.g., Y1, Y2, Y4, Y5, and Y6) and is mainly found on NPY neurons of the ARC and NTS [57,58,59,60]. Feed intake was inhibited after the administration of a selective Y2R agonist as well as direct injection of PYY3-36 into the ARC [61]. The inhibitory effect of peripheral PYY3-36 is reduced in rats treated with Y2R antagonists and abolished in knockout mice for Y2R (Table 3) [51,62]. The anorectic mechanism of PYY3-36 via IV injection can be explained as follows: PYY3-36 inhibits NPY neurons with consequent disinhibition of POMC neurons in the ARC. Additionally, hypothalamic explants incubated in vitro with PYY3-36 resulted in a decrease in NPY release and an increase in α-MSH release [51]. However, orexigenic effects by ICV injection of PYY may suggest that PYY binds Y1R and Y5R, which are mainly expressed in the hypothalamic paraventricular nucleus (PVN) [63]. Similar results were observed in studies with chickens. In chickens, PYY isolated from the small intestine did not contain PYY3-36, the major form of PYY in mammals [64]. ICV injection of PYY1–36 stimulated feed intake in neonatal chickens [65], whereas PYY3-36 administration via IV injection inhibited feed intake in neonatal chickens [66]. These inconsistent results may be attributed to the differences in doses and injection routes. Moreover, the main sites where PYY mRNA is expressed are different depending on the species [67]. In broilers, sites where PYY mRNA is mainly expressed are present in the small intestine, while sites where PYY mRNA is expressed are located mainly in the large intestine in mammals [67,68]. Because most of the studies have been performed with the administration of exogenous PYY [69], further research is needed to determine how to exert the endogenous PYY in animals including avian species.

### 4.3. Glucagon-like Peptide (GLP)

GLP is released in response to food intake from the L-cells of the small intestine and colon, the neurons in the NTS of the brainstem, and the α-cells of the Islets of Langerhans [70,71,72]. Also, pre-proglucagon, a precursor of GLP, is converted to glucagon, glucagon-like peptide-1 (GLP-1), glucagon-like peptide-2 (GLP-2), and oxyntomodulin (OXM), depending on the sites of syntheses by prohormone convertase 1 and 2 [73]. Especially, in mammals and chickens, GLP-1 and GLP-2 brain-gut peptides are produced by cleavage of the precursor preproglucagon [74].

#### 4.3.1. Glucagon-like Peptide-1 (GLP-1)

Glucagon-like peptide-1 (GLP-1) is synthesized and secreted from the L-cells in the intestinal epithelium [75,76]. A key function of GLP-1 is to regulate blood glucose levels by increasing glucose-stimulated insulin secretion and decreasing glucagon secretion [77]. Several studies reported that GLP-1 had a negative effect on feed intake of experimental animals [74,78,79]. For example, feed intake of rats and mice was reduced by ICV and IP injection of GLP-1 [78,80,81], and increased feed intake was induced by ICV injection of the GLP-1 receptor antagonist (Table 4) [78,82,83]. Furthermore, a study on humans showed the anorectic effects of administrating GLP-1, thereby causing reduced feed intake [84]. Actually, obese subjects have shown reduced levels of GLP-1 and higher weight gain [85,86]. A central nervous system component plays a role in the regulation of GLP-1, and GLP-1 is especially affected by GLP-1 receptor (GLP-1R) [87,88]. GLP-1R is abundantly detected in the hypothalamus (i.e., the ARC). This fact may be supported by the result of a previous study reporting that ICV injection of GLP-1 increased the expression of c-fos in the ARC [89]. It means that GLP-1 has the potential to act directly on the ARC in the hypothalamus. GLP-1R is also synthesized by the POMC neurons and GLP-1R agonists that exert direct as well as indirect effects on these neurons [90,91]. GLP-1R expressed in POMC neurons also indirectly affects NPY neurons [88]. According to the results of Secher et al. [92], they observed that GLP-1R restricts the synapses of NPY/AgRP neurons by influencing GABAergic neurons. As a result, GLP-1 suppressed NPY and AgRP expression, which promotes appetite, and increased POMC expression, which alleviates appetite [93]. The anorexigenic effect of GLP-1 can be explained by incretin production. Incretin is a hormone originally secreted from the digestive system while nutrients are absorbed in the intestinal tract [76]. It also increases the release of insulin and inhibits the movement of the gastrointestinal tract, causing suppressed feed intake. In chickens and ducks, GLP-1 is released from L cells in the epithelium of the jejunum and ileum [94,95,96]. This result is in agreement with the results of studies on other species reporting that GLP-1 was secreted from L cells in the intestines of mammals [71,97]. Several studies demonstrated that the anorectic effects of GLP-1 were detected in poultry as well. GLP-1 regulates the emptying of the crop and strongly reduces feed intake in chickens [98,99]. ICV and IP injection of GLP-1 significantly reduced the feed intake in laying hens and Japanese quail, respectively [100]. On the other hand, the injection of GLP-1 via IP and ICV did not affect the feed intake of laying hens and broilers [98,101]. GLP-1 levels in the blood did not fluctuate for fasting and refeeding periods in broilers [102]. In chickens, the GLP-1 receptor mRNA is widely detected in the brain as well as the gastrointestinal tract [103]. Although the role of GLP-1 in the regulation of feed intake in chickens has not been elucidated yet, the anorectic effect of GLP-1 in chickens is considered to be similar to the mechanism mentioned above in other species. It is necessary to conduct further research to determine the physiological importance of GLP-1 among various gut hormones in birds.

#### 4.3.2. Glucagon-like Peptide-2 (GLP-2)

In the L cells of the intestine, GLP-2 is co-encoded and co-stored with GLP-1 by the gene that encodes proglucagon [104]. GLP-2 has been known to play a physiological role in mammals as an intestinal growth factor [74,105]. GLP-2, like GLP-1, has various actions in distal tissues, such as regulation of appetite, inhibition of gastric emptying, and reduction of bone resorption [106,107,108]. The process of gastric emptying is critical for regulating short-term intake and may be used to modulate appetite. Previous studies reported that the anorexigenic effects of GLP-2 are closely related to gastric emptying (Table 5) [109]. Also, these actions are regulated by GLP-2 receptors that are present in the gut and brain [110]. Likewise, GLP-2 suppressed feeding behavior and activated POMC neurons in experimental animals when administered via ICV and IP [111,112,113,114]. In chickens, Honda et al. [115,116] found that central and peripheral injection of GLP-2 significantly suppressed feed intake. Moreover, Kewan et al. [117] reported that ICV injection of GLP-2 significantly improved POMC levels in the hypothalamus in layers. These findings suggest that GLP-2 exerts anorexigenic effects as GLP-1. However, another study reported that GLP-2 injection via ICV and IP did not affect feed intake and body temperature in Japanese quail [100]. GLP-2 regulation of feed intake in chickens has also not been explained yet and led to different results. Therefore, judging by these results, the anorexigenic effects of GLPs may differ depending on various factors, including animal species, dosage, and injection location of exogenous GLP-2. Further studies are required to elucidate the relationship between GLP-2 and anorexigenic effects.

### 4.4. Oxyntomodulin (OXM)

OXM is derived from the cleavage product of preproglucagon processed in endocrine L-cells of the intestine and CNS. After meal ingestion, OXM is co-secreted with the hormones GLP-1 and PYY3-36 [118]. Previous studies reported that the ICV and intranuclear injection of OXM reduced feed intake in rats (Table 6) [119,120]. In humans, OXM via intravenous (IV) injection caused reduced energy intake by 19.3%, and pre-prandial injection in obese subjects decreased body weight gain by 1.8 kg [121,122]. These findings supported that OXM is well established as a gut hormone having anorectic effects in rodents as well as humans. Although the anorectic mechanism of OXM has not been clearly elucidated, there are some hypotheses on the suppression of appetite as follows. The anorectic mechanism of OXM may be similar to that of GLP-1. Previous studies reported that the appetite suppression of OXM via IP injection was restricted when GLP-1 receptor antagonist exendin (9–39) was injected in the ARC and when the GLP-1 receptor was eliminated in mice [120,123]. Moreover, other studies have shown that OXM has an incretin effect, meaning that it multiplies glucose-dependent insulin secretion like GLP-1 [124,125]. Additionally, the anorectic mechanism of OXM may be explained by the reduction of ghrelin in blood. The administration of OXM showed a reduction of ghrelin by 44% and 15–20% in humans and rodents, respectively [120,121], suggesting that OXM may indirectly inhibit feed intake by reducing circulating ghrelin levels. In studies with chickens, OXM inhibited not only feed intake but also water intake in broilers and Japanese quail [126,127,128]. In laying hens, ICV injection of OXM dramatically reduced feed intake and increased plasma glucose concentration [127]. In addition, peripheral and intrahepatic glucose injection enhanced plasma glucose levels while suppressing feed intake in layer chicks and cockerels [129,130,131]. At least in the early post-hatch period, OXM and GLP-1 likely suppress feed intake through different mechanisms.

### 4.5. Cholecystokinin (CCK)

It was also discovered that cholecystokinin (CCK) affects appetite [132]. CCK is widely distributed across the gastrointestinal tract but is mainly synthesized in the I-cells of the duodenum and jejunum [133]. CCK is a local regulator in the gastrointestinal tract that stimulates gallbladder contraction and pancreatic enzyme secretion and inhibits gastric emptying (Table 7) [134,135]. Within 15 min of starting a meal, plasma CCK levels rise [134]. Studies in humans have shown that CCK reduces food intake and meal size [136]. Additionally, CCK has also been evaluated for its therapeutic potential in the treatment of obesity. CCK-A may be a more important receptor in the regulation of food intake and is found in the pancreas, vagal afferent, efferent neurons, nucleus of the solitary tract, and area postrema in the brainstem [137]. There has been evidence that abdominal or gastric vagotomy can block the satiety effect of peripherally administered CCK, suggesting that the CCK-A receptors on the vagus nerve may play an important role in the effect of CCK on food intake [138]. Actually, the administration of a CCK-A antagonist inhibited the reduction of feed intake by CCK in rats [139,140]. This finding is similar to the results of studies with chickens. For example, previous researchers reported that the administration of CCK in chickens restricted feed intake [141] and suppression of CCK-A receptors increased growth and body weight [142]. However, the difference between other species and poultry is the location where CCK is expressed. In poultry, CCK is released from the small intestines of chickens [143] and from the small and large intestines of ducks [144,145]. Despite the distal small intestine being the primary production site for CCK mRNA in poultry, CCK is abundant in mammal’s proximal small intestines [2,146]. CCK receptors are also located in the brain and peripheral tissues like in other species [147,148].

### 4.6. Gastric Inhibitory Polypeptide (GIP)

GIP was first reported in the study with dogs and is a 42 amino acid hormone. It is secreted from the K cell in the proximal small intestine (duodenum and jejunum) after the uptake of nutrients, including glucose, peptide, and fat [149,150]. In addition to glucagon-like peptide, GIP is classified to act as an incretin and can regulate feed intake (Table 8) [151,152]. Previous studies showed that IP and ICV injection of GIP decreased body weight gain and feed and water intake compared with mice challenged with saline [81,153]. These results clearly suggest that GIP is reduced during the hungry stage. However, there are not enough studies to elucidate the mechanisms of GIP and reduced feed intake in avian species. Therefore, further research is needed to determine whether GIP suppresses feed intake in avian species.

### 4.7. Serotonin (5-Hydroxytrptamine, 5-HT)

About 95% of serotonin is secreted via the enterochromaffin (EC) cells in EECs. The function of serotonin is different depending on its central and peripheral sites because it does not pass into the blood-brain barrier [154]. Central serotonin plays a role in regulating food consumption by increasing POMC and CART expression within the ARC, thereby having anorexigenic effects [155,156]. More specifically, the anorexigenic effects of central serotonin are controlled by 5-HT receptor 2C (HTR2C). Tecott et al. [157] found that HTR2C absence in mice was associated with increased feed intake. According to a previous study, HTR2C appeared within POMC neurons, and thus increased central serotonin can stimulate POMC neurons that are involved in the suppression of appetite [158]. On the other hand, peripheral serotonin produced by L-tryptophan has orexigenic effects [159]. Numerous studies have reported that high tryptophan supplementation increased the synthesis of serotonin, thereby increasing feed intake in pigs [160,161]. Moreover, peripheral serotonin increased intestinal motility and reduced plasma leptin and adiponectin concentrations, which can decrease appetite [159]. In poultry, insufficient tryptophan has been known to decrease serotonin synthesis [162]. However, most studies have focused on stress mitigation and meat quality, but not feed intake. There are only a few studies on serotonin and feed intake in avian species. Therefore, further research is needed to determine whether serotonin suppresses feed intake in avian species.

### 4.8. Neurotensin (NTS)

NTS is released in response to nutrient ingestion, in particular to fat, regulates GI motility and pancreatic and biliary secretion, facilitates fat translocation, and acts as an incretin [163]. NTS has been reported as a hormone having anorectic effects and is co-secreted with PYY and GLP-1, which also have anorectic effects. Actually, systemic administration of PEGylated NTS, with an increased half-life, causes a sustained reduction in food intake coupled with increased hypothalamic POMC expression [164]. Moreover, other researchers reported that peripheral injection of NTS might positively influence weight reduction [165].

## 5. Regulation of Nutrient Absorption by Gut Hormone

### 5.1. Carbohydrate Absorption by EEC Hormones

**Figure 3:**
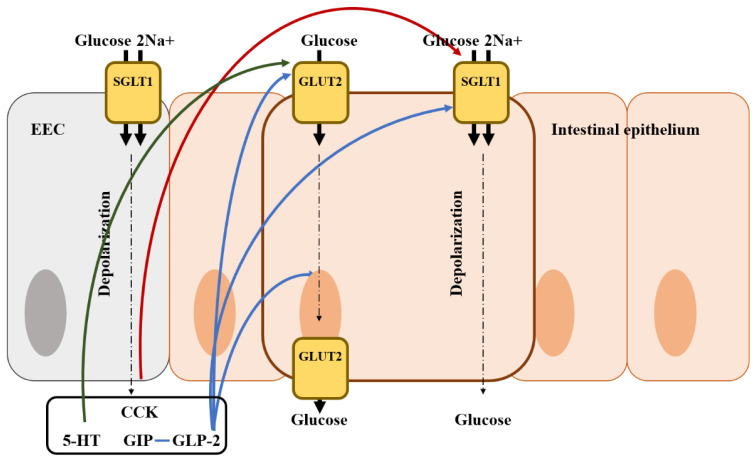
Illustration of the mode of action of glucose regulation by gut hormones from an enteroendocrine cell in the intestine and the location of the glucose transporter. Blue arrows represent that it stimulates glucose transporter and absorption; Red arrows represent that it inhibits glucose transporter and absorption; Green arrows represent that it may stimulate glucose transporter and absorption; EEC, enteroendocrine cell; CCK, cholecystokinin; GIP, gastric inhibitory polypeptide; 5-HT, 5-hydroxytryptamine (serotonin).

The absorption of nutrients by the gut is critical to stimulate gut hormone secretion by monosaccharides, peptides, and lipids [166]. EECs can actively respond to the rate at which nutrients are absorbed apart from sensing nutrients in the gut lumen [166]. Ingested carbohydrates are broken down into monosaccharides by a combination of amylases from saliva and the pancreas and hydrolases from enterocytes [167]. Glucose absorption is mainly regulated by glucose transporter (GLUT2) and sodium-glucose cotransporter 1 (SGLT1) (Figure 3). GLUT2 helps glucose absorption by translocating to the brush border in the membrane following high luminal sugar concentrations [168]. In addition, it transports glucose out of the cell by facilitated diffusion. SGLT1 is mainly used as a glucose transporter for mixed meal conditions and carries one glucose or galactose molecule as well as two Na+ ions to intestinal epithelial cells [169]. SGLT1 is expressed in EECs and absorbs two Na+ ions and glucose, leading to the stimulation of EEC hormones (GLP-1, GIP, and GLP-2) [167]. In addition, gut hormones secreted from EECs are also involved in the regulation of glucose absorption. GIP affects glucose absorption in the small intestine and increases intracellular cAMP, suggesting that GIP may up-regulate SGLT1, which is partly regulated by cAMP [170,171]. Moreover, as a result of GIP injection, GLUT2 is stimulated to release glucose to the basolateral membrane and GLUT2 translocation is increased [172,173]. GLP-2 plays a more important role in intestinal glucose absorption along with GIP [171]. Ogawa et al. [174] reported that GLP-2 up-regulates the gene expression of SGLT1 in enterocytes, resulting in an increase in the Na+/glucose transport activity. In addition, GLP-2 increases the basolateral export of GLUT2, as well as the translocation of GLUT2 to the apical brush border, thereby resulting in an increase in glucose absorption [172,175]. However, the effects of GLP-1 secreted with GLP-2 vary among previous studies. There was a reduction in the secretion of GIP and GLP-1 by mice lacking SGLT1, while the inhibition of SGLT1 enhanced GLP-1 release in SGLT1 knockout mice [176,177]. There is another gut hormone exerting negative effects on glucose absorption in the small intestine. Serotonin (5-HT) stimulates water and ion secretion in the intestine and inhibits Na+/dependent galactose absorption [178]. CCK also down-regulates the localization of SGLT1 to the brush border, thereby causing a reduction in glucose absorption [5,179]. However, there are no studies evaluating the relationship between gut hormones and glucose absorption in avian species. Therefore, further research is needed to determine the relationship among glucose transporters, gut hormones, and nutrient digestibility in avian species.

### 5.2. Fat Absorption by EEC Hormones

**Figure 4:**
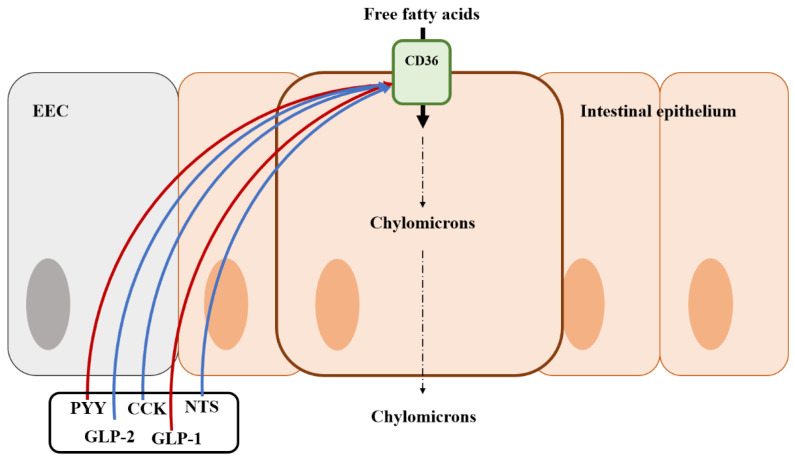
Illustration of the mode of action of fat regulation by gut hormones from an enteroendocrine cell in the intestine and the location of the fat transporter. Blue arrows represent that it stimulates the fat transporter or absorption; Red arrows represent that it inhibits the fat transporter or absorption; EEC, enteroendocrine cell; CK, cholecystokinin; GLP-1, Glucagon-like peptide 1; GLP-2, Glucagon-like peptide 2; PYY, peptide YY; NTS, Neurotensin.

Fat absorption is regulated by various gut hormones. Mice lacking CCK showed reduced weight gain and triglyceride absorption [180]. CCK is known to regulate the secretion of bile, bicarbonate, and pancreatic enzymes that play a role in emulsifying and hydrolyzing dietary fats (Figure 4) [181]. Incretins (e.g., GLP-1 and GIP) also regulate fat absorption, including an increase in insulin secretion in β-cells and a reduction of blood glucose [182,183].

Lipid transport proteins are fatty acid binding proteins and CD36, expressed in EECs, has been known as a regulator of various EEC hormones in response to fat ingestion [184,185]. Mice lacking CCK showed poor triglyceride absorption although this appeared to be independent of pancreatic enzyme secretion [5]. These findings were related to CD36 expressed in EECs. CD36 deficiency remarkably caused a reduction of CCK and secretin in experiments [184]. Therefore, CD36 can be a receptor of secretin and CCK, increasing fat absorption in the small intestine [186]. Interestingly, GLP-1 restricts the expression of CD36 but can help fat digestion via activating protein kinase A (PKA) in humans and rodents [187,188]. On the other hand, GLP-2, co-secreted with GLP-1, improves fat absorption via the up-regulation of the expression of CD36 [189]. Neurotensin (NTS) is also involved in fat absorption. Previous studies reported that NTS enhanced fat absorption by GLP-2 [190,191]. Like GLP-1, PYY has negative effects on fat absorption. The treatment of exogenous PYY to intestinal cells inhibits apolipoprotein synthesis and chylomicron formation, which are important for fat absorption, in an in vitro experiment [192]. However, unlike other nutrient regulations, there are no studies to determine the exact mechanisms of fat regulation by gut hormones.

### 5.3. Protein Absorption by EEC Hormones

**Figure 5:**
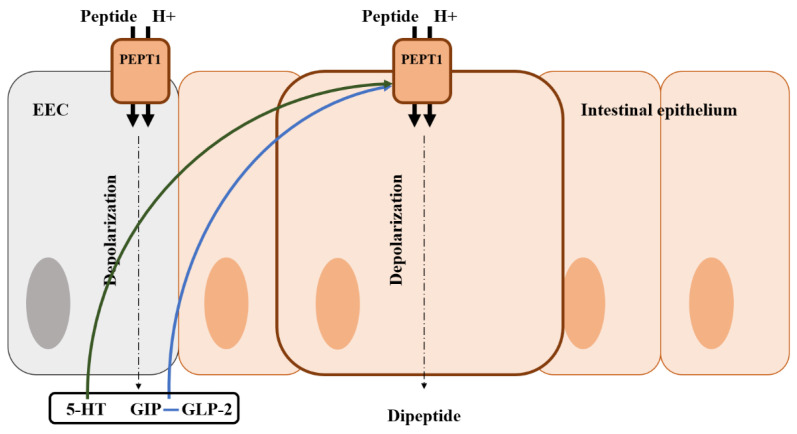
Illustration of the mode of action of amino acid regulation by gut hormones from an enteroendocrine cell in the intestine and the location of the amino acid transporter. Blue arrows represent that it stimulates the peptide transporter and absorption; Green arrows represent that it may stimulate the peptide transporter and absorption; EEC, enteroendocrine cell; GIP, gastric inhibitory polypeptide; 5-HT, 5-hydroxytryptamine (serotonin).

Ingested proteins are broken down into amino acids, dipeptides, and tripeptides by proteases in the GI tract [193]. The absorption of amino acids can be facilitated by the difference in H+ ion concentration, and peptide transporter 1 (PEPT1) is involved in most protein absorption [194]. In addition to the regulation of glucose absorption, amino acid absorption is also regulated via various gut hormones. Previous studies found that GIP improved dipeptide absorption via PEPT1 by increasing the activity of cAMP and phosphoinositide-3 kinase (Figure 5) [195]. GLP-2 also is known as a regulator of amino acid absorption [196,197,198]. This evidence could be supported by the results that GLP-2R knock-out mice had reduced amino acid uptake [199]. Conversely, GLP1, which is co-secreted with GLP-2, did not influence amino acid absorption via PEPT1 [200]. There are very few studies on protein absorption by EEC hormones compared with those on glucose and fat absorption. Therefore, it is considered that additional research is needed to determine the roles of EEC hormones on protein and amino acid absorption in poultry.

## 6. Change of Feed Intake, Nutrient Absorption and Gut Environment in Chickens under Different Stress Conditions

### 6.1. Disease

#### 6.1.1. *Eimeria* spp. Challenge

*Eimeria* spp. infects via the fecal-oral route and invades the epithelium of the intestine, thereby causing severe cell damage, diarrhea, impaired feed intake, and mortality [201,202,203]. *E. maxima* infection causes a severe reduction in feed intake due to sickness and lethargy in chickens [204]. Moreover, a meta-analysis study revealed that an *Eimeria* spp. infection decreased average daily feed intake by 20% [205]. Many studies showed that feed intake was decreased in broiler chickens challenged by *Eimeria* spp. [206,207,208]. The reduction in feed intake might be attributed to impaired nutrient absorption and intestinal cell functions by *Eimeria* infection. Chickens challenged by *Eimeria* spp. have shown severely damaged intestinal morphology, inflammation, and oxidative stress in many previous studies [209,210,211,212,213]. Chapman [202] reported that an Eimeria (e.g., *E. maxima*, *E. acervuline*, *E. mitis*, *E. tenella*) infection caused epithelial inflammation and disruption of the villi. Additionally, other researchers reported that *Eimeria* spp. oral inoculation resulted in epithelial damage of the small intestine by decreasing villus height (VH) and increasing crypt depth (CD) [214,215]. These results were attributed to the fact that *Eimeria* sporozoites and merozoites secrete proteins that can form a moving junction at the cell membrane [216,217]. By utilizing this moving junction, sporozoites and merozoites penetrate and damage intestinal epithelial cells and decrease nutrient absorption [218,219]. Previous experiments reported that *Eimeria* spp. increased ileal endogenous amino acid (AA), thereby reducing AA digestibility and decreasing ileal digestibility of dry matter, starch, and fat [202,220,221,222,223,224]. Also, an *Eimeria* infection significantly decreased the expression of amino acid and glucose transporters (e.g., APN, B°AT, b°,+ AT, EAAT3, PepT1, rBAT, GLUT2, and GLUT5) in the brush border of intestinal epithelium [223,224,225,226,227,228,229].

#### 6.1.2. Pathogen Challenge

Several studies reported that an *S. Typhimurium* infection decreased FI, thereby reducing BWG and feed efficiency [230,231,232,233,234]. These results are in agreement with those of Moharreri et al. [235] who reported that other types of *salmonella* (e.g., *S. enteritis*) also reduced total feed intake and body weight in chickens. The reduced performance observed in the challenged birds is probably due to intestinal mucosal damage induced by *S. Typhimurium* [231]. According to the results of Jazi et al. [236] and Choi et al. [234], *S. Typhimurium* led to a remarkable reduction in VH and VH: CD after challenge. These results were confirmed by previous studies, which reported *Salmonella* spp. (e.g., *S. Typhimurium* and *S. Enteritidis*) reduced VH and the VH: CD ratio in the small intestine of broilers [235,237,238]. Birds infected with *Salmonella* spp. exhibited a damaged intestinal morphological structure and reduced goblet cell numbers in the jejunum, leading to impaired absorption of nutrients along with other pathogenic bacteria [235,239]. Likewise, a *C. perfringens* infection, which is a main factor in the outbreak of necrotic enteritis (NE), and an *E. coli* infection, which is a major source of Avian colibacillosis, caused the reduction of feed intake and nutrient digestibility along with impaired intestinal morphology (e.g., reduced VH, VW and the number of goblet cells) [240,241,242].

Pathogen-induced anorexia is well known and associated with most infections [243,244]. It could be caused by the high requirements of nutrient resources to restore damage and stimulate immune responses in response to infection [243]. Thus, since most of the feed intake is controlled via the brain-gut axis by appetite-related hormones, it is considered that the reduction of feed intake by infection is inevitably caused by changes in these gut hormones. However, although these infections by pathogenic bacteria and parasites undoubtedly cause the reduction of feed intake and nutrient absorption, there are not enough studies on the relationship between infection and appetite-related hormones. Therefore, it is considered that further research is needed to understand the underlying mechanism between infection and appetite-related hormones.

### 6.2. Environment

#### 6.2.1. Heat Stress

Chickens exposed to heat stress showed significant reduction in body weight gain and feed intake [245]. Several studies reported that heat stress remarkably increased mortality and decreased feed intake in poultry [246,247,248]. Moreover, heat stress negatively affects nutrient digestibility, resulting in a reduction of DM and energy digestibility [249]. The impairment of nutrient digestibility by heat stress is supported by previous studies [250,251]. Heat stress also impaired nutrient digestibility as well as the nutrient transporters [252,253]. These findings were consistent with those of Orhan et al. [254] and Sun et al. [255], who reported that heat stress significantly decreased fatty acid binding protein (FABP) expression; binding fatty acids in the jejunum; SGLT1, which binds glucose; and PEPT1 and 2, which bind peptides in the ileum. These results were attributed to the damaged intestinal morphology, causing increased intestinal permeability to endotoxins regardless of animal species [256,257,258]. Moreover, many researchers detected that heat stress increased CD and decreased VH in the jejunum [246,247,250,259,260]. Goblet cells produce mucus to cover the intestinal epithelium, which protects it from pathogen attacks and environmental toxins. These cells also contribute to the healing of minor wounds and injuries to the epithelium. Liu et al. [260] reported that black-boned chickens exposed to heat stress showed significantly decreased goblet cell numbers in the jejunum and ileum. Similarly, Zhang et al. [261] showed that heat stress reduced the number of goblet cells and the mRNA level of the mucin-2 gene in the jejunum. Although we may expect that appetite-related hormones would not be secreted normally as various stresses damage the intestine, He et al. [262] and Wang et al. [263] found that heat stress increased CCK concentration in the serum and jejunum. On the other hand, He et al. [264] also found that heat stress caused increased ghrelin, which upregulates appetite along with CCK, which downregulates appetite in broiler chickens. Therefore, further research is needed because of these inconsistent results and there are not enough studies on the relationship between heat stress and appetite-related hormones.

#### 6.2.2. Stocking Density

High stocking density resulted in a reduction in feed intake of broilers [265,266,267,268]. High stocking densities may cause reduced feed intake due to the high environmental temperature and the reduced airflow at the bird level [269]. Similar results have been reported by Uzum and Toplu [270], showing that birds housed at high stocking density were not able to effectively dissipate their body heat to the environment, resulting in feed intake reduction for maintenance of body homeostasis. Additionally, VHs in the duodenum, jejunum, and ileum were decreased when broilers were reared at high stock density [271,272]. Moreover, stress under high stock density induced the disruption of mucosal tight junction [273]. However, although high stock densities undoubtedly cause the reduction of feed intake, there are no studies on the relationship between high stocking density and feed intake hormones; thus, further research is needed.

## 7. Conclusions and Future Perspectives

Over the last three decades, there has been significant research achievement on the gut-brain axis, which has revealed a wealth of information about the role of gut hormones in the regulation of appetite and nutrient absorption in mammals and rodents. According to the increasing evidence of EECs, EECs have been found to directly affect the regulation of appetite and nutrient absorption. Many scientists have agreed that endocrine hormones released from EECs circulate in the blood and act on CNS targets in rodents and mammals including humans. Likewise, some studies have been conducted to evaluate the appetite regulation system of chickens in recent years. The physiological roles of PYY, CCK, GLP-1, and GLP-2 have been studied in chickens like other species. In other species, hormones released from EECs have also been reported to have significant effects on nutrient absorption, and the EEC number is changed by intestinal inflammation [274]. EEC cells were increased in some species such as fish, lamb, pigs, mice, and humans during the infection [21,275,276]. Intestinal epithelial cells damaged during infection try to recover to maintain intestinal homeostasis. In particular, GLP-1 is associated with the anorexigenic effect and GLP-2 is associated with epithelial homeostasis and barrier function, and repair following injury is increased after intestinal damage [277]. In many studies, chickens under stress conditions commonly had impaired intestinal morphology, resulting in reduced feed intake and nutrient digestibility. The structural integrity of the intestine is essential for efficient digestive function and nutrient absorption, which depends on the normal development of intestinal mucosa. Therefore, nutrient digestibility and feed intake could be negatively affected if intestines are damaged and an alternation of subsequent hormones happens. However, there have been few studies on nutrient absorption and EEC hormones in chickens. Therefore, further studies are essential to elucidate the relationship between EEC hormones, appetite regulation, and nutrient absorption in chickens. In conclusion, comprehensive studies on EEC hormones are essential to understand the regulation mechanism of appetite and nutrient absorption and to determine the physiological importance of each EEC hormone in chickens under stress conditions in the future.

## Figures and Tables

**Table 1:** Overview of the different types of enteroendocrine cells, secreted peptides, and their brief function.

Cell Type	Hormone	Location	Function	Reference
X or A/ P or D1	Ghrelin	Stomach and proximal intestine	Stimulation of food intake	[18,19]
G	Gastrin	Stomach	Gut motility and pancreatic enzyme release	
D	STT	Stomach and intestine	Inhibition of GI hormone	
EC	Serotonin	Stomach and intestine	Inhibition or stimulation of food intake Stimulation of gut motility	
I	CCK	Proximal intestine	Inhibition of food intake and stimulation of the gallbladder	
K	GIP		Stimulation of insulin and gastric acid secretion, nutrient sensing	
S	Secretin		Inhibition of gastric acid secretion and motility	
N	NTS	Distal intestine	Stimulation of gastric acid and bile secretion	
L	GLP-1, GLP-2, PYY, OXM, NTS		Inhibition of food intake, gastric acid secretion, and response in glucose absorption	

Abbreviation: EC, enterochromaffin cells; STT, somatostatin; CCK, cholecystokinin; GIP, glucose-dependent insulinotropic peptide; NTS, neurotensin; GLP-1, glucagon-like peptide-1; GLP-2, glucagon-like peptide-2; PYY, peptide YY; OXM, oxyntomodulin.

**Table 2:** Effects of functions and/or effects according to dosage and location of ghrelin injection.

Species	Location	Dosage	Function/Effects	Reference
Rat	ICV	1.2 µmol/kg BW	↑ Feed intake and body weight gain	[36]
	ICV	3 nmol	↑ Feed intake, GH hormone, ACTH hormone, and TSH hormone	[29]
	IP	30 nmol	↑ Feed intake and GH hormone	
Human (Lean vs. Obese)	IV	5.0 pmol/kg BW	↑ More food intake in obese	[35]
Laying hen	IV	0.6 nmol/100 g BW	↔ feed intake and expression of mRNA in proventriculus	[42]
Broiler	IV	1.0 nmol/100 g BW	↓ Feed intake/↔ heat production	[41]
Broiler	IV	1.0 nmol/chick	↑ Feed intake for 30 min after injection, and then no effect of feed intake	[43]
Broiler	IP	0.5–2.0 nmol/100 g BW	↓ Feed intake	[44]
Broiler	IV	1 nM/100 g BW	↓ Feed intake	[46]
Japanese quail	IP	0.5–1.0 nmol/chick	↑ Feed intake	[45]
		3.0 nmol/chick	↓ Feed intake	
	ICV	0.5–1.0 nmol/chick	↓ Feed intake	

Abbreviation: BW, body weight; IV, intravascular injection; IP, intraperitoneal injection; ICV, intracerebroventricular injection; ↓, represents decreased or downregulated; ↑, represents increased or upregulated; ↔, represents no difference.

**Table 3:** Effects of functions and/or effects according to dosage and location of peptide YY (PYY) injection.

Species	Location	Dosage	Function/Effects	Reference
Rat	ICV	0–1175 pmol	↑ Feed intake	[52]
Rat	IV	0.3–10.0 µg/100 g BW	↓ Feed intake	[51]
Mice	IA			
Human	IV	0.8 pmol/kg BW	↓ Calorie intake	
Mouse	ICV	5.0 nmol/day	↑ Feed and water intake ↑ Body weight gain	[53]
Rat	IV	25 nmol/kg BW	↓ Feed intake	[56]
Broiler	ICV	59 or 118 pmol	↑ Feed intake	[65]
Broiler	IV	3–6 nmol/kg BW	↓ Feed intake	[66]

Abbreviation: BW, body weight; IV, intravascular injection; ICV, intracerebroventricular injection; IA, intra-arcuate injection; ↓, represents decreased or downregulated; ↑, represents increased or upregulated.

**Table 4:** Effects of functions and/or effects according to dosage and location of glucagon-like peptide-1 (GLP-1) injection.

Species	Location	Dosage	Function/Effects	Reference
Rat	ICV	10 µL	↓ Feed intake/↑ c-fos in PVN (Exendin (9–39) injection as GLP-1-receptor antagonist inhibits effects of GLP-1 on feed intake)	[78]
	IP	100 µg/kg BW	↓ Feed intake	[80]
Mouse	ICV	1–3 nmol/kg	↓ Feed intake and body weight ↑ POMC expression in ARC	[81]
Human (Obese)	IV	0.75 pmol/kg BW	↓ Appetite, food intake, and body weight gain ↓ Plasma glucose and Gastric emptying	[85]
Laying hens	ICV	15–60 pmol/kg BW	↓ Crop emptying/↔ Feed intake ↓ Feed intake (IP injection in unpublished paper)	[98]
	IP	120–3000 pmol/kg BW	↔ Crop emptying/Feed intake	
	ICV	1 pmol/kg of BW	↓ Feed intake and plasma glucose	[99]
	ICV	5 pmol	↓ Feed intake	[101]
Broiler	ICV	5 pmol	↔ Feed intake	
Japanese quail	ICV, IP	0.5–1.0 nmol/kg BW	↓ Feed intake and body temperature	[100]

Abbreviation: BW, body weight; IV, intravascular injection; IP, intraperitoneal injection; ICV, intracerebroventricular injection; ↓, represents decreased or downregulated; ↑, represents increased or upregulated; ↔, represents no difference.

**Table 5:** Effects of functions and/or effects according to dosage and location of glucagon-like peptide-2 (GLP-2) injection.

Species	Location	Dosage	Function/Effects	Reference
Japanese quail	ICV	0.01–1.0 nmol/kg of BW	↔ Feed intake and body temperature	[100]
	IP	0.5–5.0 nmol/kg BW		
Rat	ICV	10 µg	↓ Feed intake	[111]
Mouse	ICV	-	↓ Feed intake	[112]
Mouse	IP	0.30 µg/g BW	↓ Feed intake and gastric emptying rate	[113]
Laying hen	ICV	30–300 pmol	↓ Linearly feed intake and concentration of glucose	[115]
Broiler	ICV	30 pmol	↓ Feed intake	
Broiler	ICV	30–300 pmol	↓ Feed intake	[116]
		10 pmol		
	IP	1.5 nmol/kg BW	↓ Feed intake	

Abbreviation: BW, body weight; IP, intraperitoneal injection; ICV, Intracerebroventricular injection; ↓, represents decreased or downregulated; ↔, represents no difference.

**Table 6:** Effects of functions and/or effects according to dosage and location of oxyntomodulin (OXM) injection.

Species	Location	Dosage	Function/Effects	Reference
Rat	ICV	3 nmol	↓ Feed intake	[119]
	IPVN	1 nmol		
Rat	IP	100 nmol/kg BW	↓ Feed intake and body weight	[120]
	IA	1 nmol	↓ Feed intake	
Human	IV	3 pmol/kg BW	↓ Energy intake and ghrelin	[121]
		400 nmol	↓ Energy intake and body weight gain	[122]
Broiler	ICV	0–2.68 nmol	↓ Linearly feed intake and water intake	[126]
Laying hen	ICV	0.1–1.0 nmol	↓ Feed intake ↑ Plasma glucose and corticosterone	[127]
Japanese quail	ICV, IP	0.32–1.30 nmol	↓ Feed intake and water intake ↑ c-Fos activity in ARC	[128]

Abbreviation: BW, body weight; IV, intravascular injection; IP, intraperitoneal injection; ICV, intracerebroventricular injection; IA, intra-arcuate injection; IPVN, intraparaventricular nucleus injection; ↓, represents decreased or downregulated; ↑, represents increased or upregulated.

**Table 7:** Effects of functions and/or effects according to dosage and location of cholecystokinin (CCK) injection.

Species	Location	Dosage	Function/Effects	Reference
Human	IV	4 ng/kg BW	↓ Food intake	[136]
Rat	IP	16 µg/kg BW	↓ Feed intake	[139]
Human	IV	0.75 µg/mL	↓ food intake ↑ Food intake by CCK antagonist	[140]
Laying hen	IP	60–300 nmol/kg BW	↓ Feed intake ↑ Corticosterone by 300 nmol/kg BW in IP injection and 1 nmol ICV injection	[141]
	ICV	0.2–1.0 nmol		

Abbreviation: BW, body weight; IV, intravascular injection; IP, intraperitoneal injection; ICV, intracerebroventricular injection; ↓, represents decreased or downregulated; ↑, represents increased or upregulated.

**Table 8:** Effects of functions and/or effects according to dosage and location of gastric inhibitory polypeptide (GIP) injection.

Species	Location	Dosage	Function/Effects	Reference
Mouse	ICV	1–6 nmol/kg	↓ Feed and water intake	[81]
	IP	0.12 mg/kg	↓ body weight gain ↔ Feed intake	[153]

Abbreviation: IP, intraperitoneal injection; ICV, intracerebroventricular injection; ↓, represents decreased or downregulated; ↔, represents no difference.

## Data Availability

The data presented in this study are available in this paper.
